# Connection between Tumor Suppressor BRCA1 and PTEN in Damaged DNA Repair

**DOI:** 10.3389/fonc.2014.00318

**Published:** 2014-11-10

**Authors:** Akari Minami, Atsuko Nakanishi, Yasunori Ogura, Yasuko Kitagishi, Satoru Matsuda

**Affiliations:** ^1^Department of Food Science and Nutrition, Nara Women’s University, Nara, Japan

**Keywords:** BRCA1, PTEN, genome stability, reactive oxygen species, DNA repair, cell signaling, carcinogenesis

## Abstract

Genomic instability finally induces cell death or apoptosis. The tumor suppressor, phosphatase and tensin homolog on chromosome 10 (PTEN), is a dual-specificity phosphatase, which has protein phosphatase activity and lipid phosphatase activity that antagonizes PI3K activity. Cells that lack *PTEN* have constitutively higher levels of PIP3 and activated downstream PI3K/AKT targets. BRCA1, a well-known breast cancer tumor suppressor, is to associate with breast cancer risk and genetic susceptibility. Many studies have demonstrated that PTEN, as well as BRCA1, plays a critical role in DNA damage responses. The BRCA1 functionally cooperates with PTEN and might be an essential blockage in the development of several tumors. Actually, the *PTEN* and *BRCA1* genes are recognized as one of the most frequently deleted and/or mutated in many human cancers. The PI3K/AKT pathway is constitutively active in BRCA1-defective human cancer cells. Loss or decrease of these PTEN or BRCA1 function, by either mutation or reduced expression, has a role in various tumor developments. This review summarizes recent findings of the function of BRCA1 and PTEN involved in genomic stability and cancer cell signaling.

## Introduction

**Figure 1:**
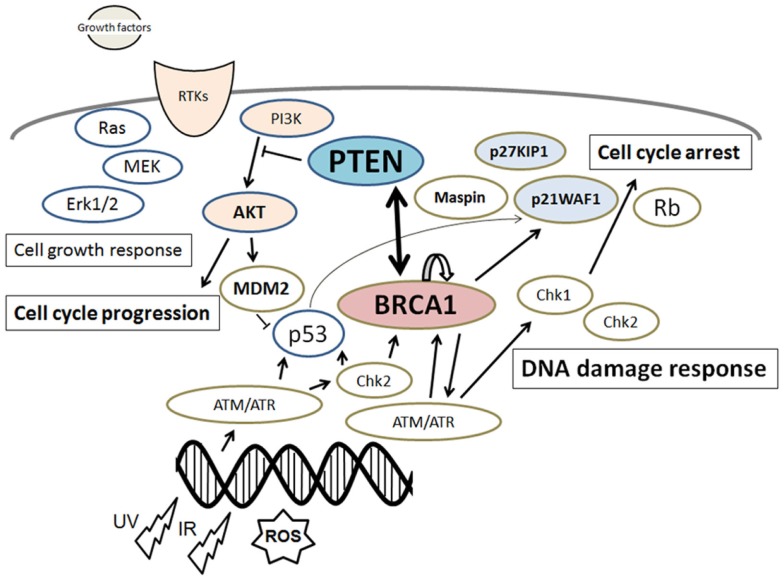
**Schematic depiction of the integrative model of tumor suppressors signaling including PTEN and BRCA1**. Examples of molecules known to act on DNA damage response, cell proliferation, and cell cycle via the regulatory pathways are shown. Note that some critical pathways have been omitted for clarity.

Germline mutations in the breast cancer susceptibility gene 1 (BRCA1) extensively increase the risk of breast and ovarian cancers (1, 2). BRCA1-related tumorigenesis may be mainly caused by increased DNA damage and decreased genome stability that is a major hallmark of cancer (3). To maintain genomic integrity, cells are equipped with committed sensors to monitor DNA repair and/or to impose damaged cells into apoptotic cell death (4). Although functional roles of BRCA1 may include the regulation of DNA damage repair, cell cycle progression, and maintenance of genomic integrity, the precise function of the BRCA1 gene as a tumor suppressor is still not clear. It has been shown that BRCA1 deficiency activates the AKT oncogenic signaling pathway (5). Also, activation of the phosphoinositide 3-kinase (PI3K) is often associated with the BRCA1-related breast cancers in clinical sample (6). The PI3K/AKT pathway might have an essential role in the proliferation of malignant tumor cells related to the BRCA1 functions (Figure 1). BRCA1 can downregulate AKT activation via the direct physical interaction (5, 7). In addition, AKT activation inversely correlates with the BRCA1 expression in human breast cancers (8). Moreover, BRCA1 negatively regulates the PI3K/AKT pathway in breast cancer cells (9). Phosphatase and tensin homolog on chromosome 10 (PTEN) is a dual protein/lipid phosphatase that inhibits the PI3K/AKT pathway, whose inhibition eventually reduces cell growth and cell proliferation (10, 11). The PTEN is also a tumor suppressor molecule and seems to protect from bad prognosis of several cancers. In other words, absence of PTEN worsens prognosis in early stages of cancer (12, 13). Furthermore, germ-line mutations of *PTEN* are the cause of *PTEN* hamartoma tumor syndromes (Cowden syndrome, Bannayan-Riley-Ruvalcaba syndrome, *PTEN*-related Proteus syndrome, Proteus-like syndrome) with increased risk for the development of cancers (14). The PTEN has been shown to be involved in an intricate network of interactions with other molecules (Figure 1). In this review, we summarize the current research and our view of how PTEN and BRCA1 function with their partners to transduce signals downstream and what are the implications for cancer-associated biology.

## Characteristics of BRCA1 and Genome Instability

Because BRCA1 may play an essential role in maintaining genome stability, the mutation of BRCA1 is associated with increased genomic instability in cells (15), which consequently accelerates the mutation rate of other critical genes. Actually, studies have established functional roles for BRCA1 in DNA damage signaling, DNA repair processes, and cell cycle checkpoints (16, 17). In addition, inherited BRCA1 germline mutation revealed a genetic susceptibility leading to high risk of breast and ovarian cancers (18, 19). It has been identified that common variation in BRCA1 gene is also associated with prostate cancer (20). Increased prostate cancer risk and an aggressive clinical course have been reported for BRCA1 mutation (21). Furthermore, several important prostate cancer targets are modulated by BRCA1 (22). *BRCA1* cDNA encodes for 1863 amino acids protein with two putative nuclear localization signals and an amino terminal conserved RING finger motif, which is the most common motif present in E3 ubiquitin ligases. The RING finger domain interacts with E2 ubiquitin ligases and exerts maximal E3 ligase activity (23). Knock-in mice with deficient BRCA1 RING finger mutant display diverse genomic instability and tumor-forming phenotypes (24). The carboxyl-terminal domain of BRCA1 is involved in association with specific phosphorylated proteins. BRCA1 itself becomes hyper-phosphorylated after exposure to the DNA damaging agents, and the specific function of BRCA1 seems to be regulated by the phosphorylation (25, 26). Exon 11 encodes a largely unstructured region of the BRCA1 protein that is phosphorylated by the ATM and Chk2 kinases in a DNA damage-dependent manner (27, 28). Principally, the main DNA damage recognition molecule may be the ATM, which is a checkpoint kinase that phosphorylates a number of proteins including BRCA1 and p53 in response to DNA damage (29). Inhibition of DNA repair pathway seems to block the mechanisms that are also required for cell survival in the presence of oncogenic mutations.

Several functions of BRCA1 including roles in the DNA repair may contribute to its tumor suppressor activity. Although BRCA1 gene mutations are rare in sporadic breast and/or ovarian cancers, BRCA1 protein expression is often reduced in the sporadic cancer specimens. The BRCA1 has the important role in concert with Rad50 and Rad51, a DNA recombinase related to the bacterial RecA protein, for the genome stability (30). Phosphorylation status of BRCA1 in response to DNA damage controls the selectivity of DNA repair events, and the function of BRCA1 seems to be regulated by this phosphorylation (30). It has been reported that Chk2 kinase and its downstream target BRCA1 have been functionally linked to the DNA damage response pathway (31). In addition, BRCA1 with the Chk2-mediated phosphorylation is also associated to the cellular spindle formation and chromosomal stability (31). The DNA repair system strictly maintains genomic fidelity through the recognition and repair of the damaged nucleotides. Genetic defects in DNA damage response genes and/or downregulation of the DNA repair mechanism certainly promote genomic instability, which can lead to carcinogenesis. Therefore, cells are equipped with multiple DNA repair mechanisms to the preservation of genomic stability (32). Basically, the role of BRCA1 in cell cycle control has been understood by its ability to interact with various cyclins and various cyclin-dependent kinases (33, 34). The BRCA1 activates the CDK inhibitor p21WAF1 and the p53 tumor suppressor protein, which regulates several genes that control cell cycle checkpoints (33, 34). Given the significant importance of the BRCA1 network in all proliferating cells, insights into the underlying mechanisms of BRCA1 function on chromatin might extend beyond hereditary cancers. Understanding such mechanisms of genome maintenance leads to an improved therapies that target DNA repair deficiency in a variety of malignancies. Hence, the regulation of DNA repair levels may be an innovative therapeutic modality in certain cancers. Either survival or apoptosis, which is determined by the balance between DNA damage and DNA repair levels, may raise the major problems in cancer therapy at that time (35).

## Characteristics of PTEN and Cancer

*Phosphatase and tensin homolog on chromosome 10* tumor suppressor gene is frequently deleted and mutated in various human cancers. Such many somatic PTEN mutations and loss of heterozygosity in cancer at the *PTEN* locus implicate a key role for PTEN in the etiology of various cancers (36, 37). Human genomic *PTEN* gene locus on chromosome 10q23.3 contains 9 exons encoding a 5.5 kb mRNA that has a 403 amino-acid open reading frame (38, 39). The *PTEN* gene is ubiquitously expressed throughout early embryogenesis in almost mammals (40). The PTEN enzyme prefers acidic phospholipid substrates such as PIP3 that is the principal second messenger of the PI3K pathway. The PI3K mediates receptor tyrosine kinase signaling to the survival kinase AKT (Figure 1). PTEN depressingly regulates the activity of PI3K/AKT signaling over converting phosphatidylinositol 3,4,5-triphosphate (PIP3) into phosphatidylinositol 4,5-bisphosphate (PIP2). PTEN might act as a regulator of keeping basal levels of PIP3 below a threshold for the signaling pathway activation. The PTEN inactivation is often involved in the carcinogenesis of some cancers (38), which causes an increase in cellular PIP3 levels. Subsequently, activated PI3K/AKT signaling causes increased expression of several genes for cell growth, cell survival, and cell migration, which are all critical for tumor development (39, 40). Remarkably, some of rosemary extracts may inhibit *PTEN* expression in K562 culture cells (41). PTEN can be controlled by posttranslational regulation including phosphorylation, acetylation, methylation, oxidation, and so on (42, 43). Because PTEN may be regulated by ubiquitin-mediated proteasomal degradation, a common mechanism to control protein levels, insecurity of PTEN correlated with some of its mutations has been shown to comprise protein interactions (44). Casein kinase 2 mediated phosphorylation stabilizes PTEN protein in an inactive state by inhibiting its proteasomal degradation (45, 46). Therefore, inhibition of the PTEN phosphorylation by the Casein kinase 2 results in enhanced PTEN activity and a subsequent suppression in AKT function (45, 46). Overexpression of PTEN induces growth inhibition by supporting cell cycle arrest, which needs lipid phosphatase activity of PTEN (47, 48). Overexpression of PTEN also correlates with decreased levels and nuclear localization of cyclin D1 (49, 50), a key cell cycle molecule regulated by AKT kinase. One of the mechanisms by which PTEN induces cell cycle arrest is by regulating AKT function so that levels of the cell cycle inhibitor p27KIP1 is increased (51, 52). Despite the central role of PTEN as a negative regulator of the PI3K pathway has been revealed, studies have reported that tumor suppressive activities of PTEN are exerted from within the nucleus, where catalysis of PIP3 does not seem to be present at least a dominant function of the enzyme (53, 54). Nuclear localization of PTEN seems to mediate tumor suppressive activities independent of the AKT pathway through inhibiting anchorage-independent growth (53, 54). The PTEN activities in nucleus may contain the regulation of gene expression and genomic stability (53, 54).

Several growth factor-activated AKT signaling pathway promotes progression of cell cycles by acting on downstream factors involved in controlling the G1/S and/or G2/M transitions (55). Studies have also implicated AKT kinase in modifying the status of genome stability and response for DNA damages (55). In addition, PTEN plays a critical role in damaged DNA repair through its interaction with ATM-p53 pathways in an AKT-independent manner (56). The upregulation of PTEN represses AKT and MDM2 activity, which enhances the level of p53, thereby inducing G2/M arrest and apoptosis (57, 58). In addition, it has been suggested that nuclear PTEN plays a distinctive role to protect cells upon oxidative damage (59). One mechanism by which reactive oxygen species (ROS) are thought to employ its effects may be through the regulation of target molecules including several kinases, PI3K, AKT, and PTEN (60). Actually, the catalytic activity of PTEN can be modulated by the ROS, and cellular PTEN activity is also repressed by the oxidative stress (60, 61). In addition, endogenous oxidant production in macrophages inactivates a fraction of the cellular PTEN (62, 63). It has been reported that ROS levels are increased in the retinal pigment epithelium cells in association with phosphorylation and inactivation of PTEN (64, 65). Phosphorylated inactivation of the PTEN and the consequent AKT activation in cells are withdrawn by antioxidant treatment. ROS mediates PTEN inactivation but ROS does not affect the PTEN expression. Hence, the uncontrolled generation of ROS might contribute to cell proliferation and tumor growth by inhibiting the PTEN function.

## Functional Interplay between BRCA1 and PTEN in Breast Cancer

Several PI3K inhibitors favorably reduce proliferation of BRCA1-defective breast cancer cells. BEZ235 inhibits not only PI3K/mTOR but also ATM/ATR and some of DNA-dependent protein kinases with similar effectiveness *in vitro* (66, 67). It is possible that ATM pathways are involved in upregulation of the PI3K/AKT pathway in BRCA1-defective cancer cells. Perifosine, a PI3K/AKT inhibitor, prevents translocation of AKT from the cytoplasm to the plasma membrane by targeting the pleckstrin homology (PH) domain, thereby preventing phosphorylation of AKT by upstream kinases (68). Perifosine prevents proliferation of breast cancer cell lines in a BRCA1-dependent manner (9). Remarkably, combination of PI3K pathway inhibitors with chemotherapeutic drugs such as doxorubicin, cisplatin, or topotecan results in enhancing cancer cell killing properties in BRCA1-defective breast cancer cells (69, 70), suggesting that the PI3K/AKT pathway may be activated in BRCA1-defective breast cancer cells. Hence targeting this PI3K/AKT pathway in combination with chemotherapeutic agents is a plausible strategy for treatment of certain cancer cells. Importantly, it has been shown that depletion of AKT significantly reduces tumor formation induced by *Brca1* deficiency in the KO mice (8). On the other hand, AKT activation promotes the expression of BRCA1. In addition, phosphorylation of BRCA1 by AKT increases total BRCA1 protein expression by preventing proteasomal degradation (7). However, it has also been reported that AKT phosphorylation has an inverse correlation with BRCA1 expression in human breast cancers (71, 72). Phosphorylation site in BRCA1 by AKT is at S694 of BRCA1 (7). AKT activation also appears to support nuclear localization of BRCA1, and co-expression of activated AKT with intact BRCA1 decreases radiation sensitivity (7), suggesting this interaction has functional consequences for BRCA1 function in DNA repair.

In contrast, BRCA1 may regulate the PI3K/AKT pathway by acting on upstream kinases of AKT. For example, overexpression of wild-type BRCA1 could further reduce basal phosphorylation (S473/T308) of AKT levels in MCF7 cells. Transient expression of wild-type BRCA1 also abolished the phosphorylation of AKT (S473/T308) in PTEN negative cells (9, 73). Negative mutations and/or decreased expression of the *BRCA1* gene may thus activate the PI3K/AKT cancer proliferation pathway (5). In addition, BRCA1 may directly downregulate the AKT protein either by ubiquitin-mediated proteasomal degradation or by activating a protein serine/threonine phosphatase PP2A in breast cancer cells (5). *BRCA1* mutant cells accumulate nuclear phosphor-AKT and subsequently inactivate the transcriptional activity of FOXO3a, a central nuclear target of the phosphor-AKT (74, 75). Significantly, some of breast cancers with BRCA1 mutations have high frequencies of PTEN mutations (76), and the resulting PI3K/AKT activation induces the growth of those cancers (77). PTEN loss is highly associated with BRCA1 breast cancers, which could result from genome instability involving homozygous deletions, DNA double-strand breaks and so on (76). Interestingly, PTEN loss is not observed in estrogen hormone receptor-positive BRCA1-associated tumors (76). Loss of PTEN expression might be a starting event in a variety of BRCA1-associated cancers (78). Nuclear PTEN might affect a variety of biological functions and plays a role in DNA repair, cell cycle arrest, and genome stability with BRCA1. In that case, PTEN acts on chromatin and regulates expression of Rad51, which reduces the incidence of spontaneous double-strand breaks (79, 80). Several reports have indicated that reduced levels of PTEN are associated with radioresistance, which can be suppressed by the ectopic PTEN expression (81, 82).

## Perspective

**Figure 2:**
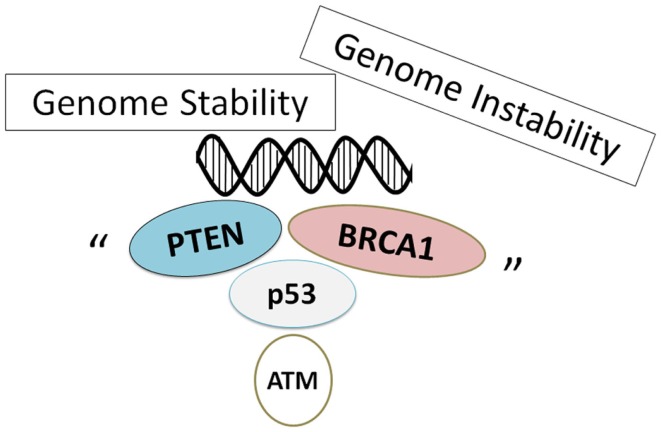
**Schematic illustration implying that genome stability is sustained on several tumor suppressors**. Note that some critical other functions have been omitted for clarity.

Genome stability might be sustained on several tumor suppressors (Figure 2). Loss of PTEN increases cell survival and reduces DNA repair, which may lead to genomic instability and may enhance radiosensitivity. In case of cancer cells that compromise therapeutic success, targeting inhibition of PTEN-related PI3K/AKT/mTOR pathway has been shown to prevent tumorigenesis and progression. Indeed, for example, rapamycin, an mTOR-specific inhibitor, prevents leukemia development in PTEN-null mouse models (83, 84). However, the effectiveness of rapamycin may require PTEN deletion or genetic loss of PTEN function. The presence of wild-type PTEN may compromise the efficacy of rapamycin (85). In addition, studies show that mTOR inhibition decreases PTEN transcription and subsequently activates AKT (85). Further detailed mechanistic understanding of the roles of PTEN in DNA repair and DNA damage response in different tissues and cell types will help us fully understand the precise molecular mechanisms by which PTEN maintains genomic stability and contributes to tumor suppression and therapeutic efficacy. PTEN and BRCA1 may be regulated and interact each other at multiple levels including transcription, protein modulation, and protein stability. Understanding the connection between tumor suppressor BRCA1 and PTEN would facilitate the development of effective agents and strategies to better treatment against cancer. The PTEN inhibitor has been shown to effectively activate primordial follicles both in neonatal mouse ovaries and in human ovarian cortical tissues (86, 87). It is important to investigate the functional linkage between PTEN and BRCA1 in those ovarian samples, and elucidation of interaction-specific functions may provide insight into regulatory aspects of these tumor suppressors as well as opportunities for therapeutic intervention. Indeed, the regulation is crucial for the effective design of novel ovarian cancer therapeutics. Further mechanistic studies are needed in order to understand the precise molecular mechanisms for the effective treatment of cancers with PTEN/BRCA1 signal alterations. Targets within this pathway could provide strategies for modulation of PTEN/BRCA1 proteins, which may prove therapeutically beneficial for breast, ovarian, and prostate cancer treatment.

## Conflict of Interest Statement

The authors declare that the research was conducted in the absence of any commercial or financial relationships that could be construed as a potential conflict of interest.

## Abbreviations

ATM, ataxia telangiectasia mutated; BRCA1, breast cancer susceptibility gene 1; LOH, loss of heterozygosity; MDM2, murine double minute 2; NLS, nuclear localization signal; PTEN, phosphatase and tensin homolog deleted on chromosome 10; PIP3, phosphatidylinositol 3,4,5-triphosphate; PIP2, phosphatidylinositol 4,5-bisphosphate; PI3K, phosphoinositide-3 kinase; RING, really interesting new gene finger domain; ROS, reactive oxygen species.
